# Intermittent high glucose-induced oxidative stress modulates retinal pigmented epithelial cell autophagy and promotes cell survival via increased HMGB1

**DOI:** 10.1186/s12886-018-0864-5

**Published:** 2018-08-06

**Authors:** Wei Zhang, Jian Song, Yue Zhang, Yingxue Ma, Jing Yang, Guanghui He, Song Chen

**Affiliations:** Tianjin Eye Hospital, Tianjin Key Lab of Ophthalmology and Visual Science, Tianjin Eye Institute, Clinical College of Ophthalmology Tianjin Medical University, No. 4, Gansu Road, Tianjin, 300020 China

**Keywords:** Intermittent high glucose, HMGB1, Oxidative stress, Autophagy, Retinal pigment epithelium cell

## Abstract

**Background:**

In this study, we evaluated the effects of intermittent high glucose on oxidative stress production in retinal pigmented epithelial (RPE) cells and explored whether the mechanisms of autophagy and apoptosis in oxidative stress are associated with high-mobility group box 1 (HMGB1) protein.

**Methods:**

Cultured human RPE cell line ARPE-19 cells were exposed to intermittent high glucose-induced oxidative stress. Reactive oxygen species (ROS) was determined by 2′, 7′-dichlorofluorescin diacetate (DCFH-DA); and malonyldialdehyde (MDA), superoxide dismutase (SOD) by commercial kits. Transmission electron microscopy was used to observe the generation of autophagosome. And MTT assay was used to examine the effect of autophagy on cell viability. For the inhibition experiments, cells were pre-incubated with lysosomal inhibitors NH4Cl or N-acetyl cysteine (NAC).Western blot was used to measure the expression patterns of autophagic markers, including LC3 and p62. The expression of HMGB1 was detected by immunohistochemistry.Cells were pre-incubated with HMGB1 inhibitor ethyl pyruvate (EP) ,then detected the expression pattern of autophagic markers and level of cellular ROS.

**Results:**

We found that intermittent high glucose significantly increased oxidative stress levels (as indicated by ROS, MDA, SOD), increased in the generation of autophagosome, decreased the level of p62, induced conversion of LC3 I to LC3 II. We further demonstrated that the NH4Cl/NAC inhibited intermittent high glucose-induced autophage by altered level of LC3 and p62. Intermittent high glucose-induced autophagy is independent of HMGB1 signaling, inhibition of HMGB1 release by EP decreased expression pattern of autophagic markers and level of cellular viability.

**Conclusions:**

Under intermittent high glucose condition, autophagy may be required for preventing oxidative stress-induced injury in RPE. HMGB1 plays important roles in signaling for both autophagy and oxidative stress.

## Background

Diabetic retinopathy (DR) is the main cause of visual loss in the adults. Increased retinal inflammatory cytokines are closely related to retinal pathologies in DR. The injury and cell apoptosis of retinal pigment epithelial (RPE) cells are considered to be happened in DR. RPE is a monolayer of pigmented cells that separates the neural retina from a network of fenestrated vessels called the choriocapillaris, which serves as the major blood supply for the photoreceptors, and therefore the RPE constitutes the outer blood-retinal barrier (BRB). Impairment of the outer BRB is increasingly recognized to play an important role in the initiation and progression of early DR. [1, 2] Oxidative stress and impaired protein degradation in RPE cells may result in RPE damage and dysfunction [3]. Although the mechanism of RPE cells injury induced by diabetes is not yet clear, studies show that fluctuating glucose is more harmful to RPE cells than constantly high glucose concentration [4, 5]. Furthermore, fluctuating glucose promotes a greater increase in inflammatory cytokine production from retinal endothelial cells than constantly high glucose through release of reactive oxygen species (ROS) which is another important trigger for DR pathogenesis [6]. In addition, ROS can further exaggerate inflammation in the pathogenesis of DR.

Autophagy is a process of catabolic reaction that involves the mechanical degradation of cellular components through lysosomes [7]. Autophagy plays a key role in the growth, development, and homeostasis of cells by maintaining the balance between the synthesis, degradation, and recirculation of cellular components [8]. Autophagy is also the key to RPE homeostasis because the RPE has high metabolic activity under a highly oxidative environment. ROS can induce autophagy through several different mechanisms including catalase, autophagy related gene 4 (ATG4) [9]. Therefore, the damaged autophagy or lysosome activity may lead to insufficiently remove the intracellular organelles or protein aggregates of oxidative damage, which leads to the accumulation of toxic substances within and outside the cells and damages the RPE function during DR.

Thus, autophagy could be regulated and executed, which is crucial for maintaining cellular homeostasis, as a key adaptive mechanism against multiple cellular stress situation. However, the function of autophagy in RPE is remain unclear on glucose fluctuation stress. Moreover, we recently demonstrated that oxidative stress is implicated in retinal inflammation during DR. [10] In this study, we evaluated the effects of intermittent high glucose on oxidative stress production in RPE cells and explored whether the mechanisms of autophagy and apoptosis in oxidative stress are associated with high-mobility group box 1 (HMGB1) protein.

## Methods

### Cell culture

Human cell line, ARPE-19 cells was obtained from the American Type Culture Collection. The cells were cultured in DMEM medium containing 10% Foetal bovine serum (FBS) and 1% penicillin/streptomycin. ARPE-19 cells were chosen as monolayers, they express all the signature genes of human RPE cells. Cells were exposed to the following experimental conditions for 48hs: 1) normal glucose (5 mM, Control); 2) constant high glucose (25 mM, HG); 3) normal and high glucose alternating every 3 h (HG-Int). Medium were collected and cells harvested for analysis.

### Proliferation assay

Proliferation was analysed by MTT (Becton Dickinson, Bedford, MA, USA) as previously described [11]. In short, we seeded ARPE-19 cells in 96-well plates with 6 × 10 [3] cells/well and cultured them at 37 °C with 5% CO_2_ for 24 h. Second, added 10 μl of MTT solution (5 mg/ml) to each well, incubating ARPE-19 cells for 4 h at 37 °C. After the formation of a crystal, discarded MTT medium and replaced with 150 μl of dimethyl sulfoxide (DMSO) (Sigma Chemical Co., USA) to dissolve crystal. Then, the plates were shaken for 5 min. The absorbance of each well was recorded by a microplate spectrophotometer at 570 nm.

### Detection of reactive oxygen species (ROS)

The concentration of ROS in ARPE-19 cells was detected by measuring the fluorescent signal from the DCFDA (redox-sensitive-fluoroprobe- 2′, 7′-dichlorofluorescein-diacetate). In short, ARPE-19 cells were cultured in phenol red-free DMEM in 12-well plates, and then incubated with the following experimental conditions: 1) normal glucose (5 mM, Control); 2) constant high glucose (25 mM, HG); 3) normal and high glucose alternating every 3 h (HG-Int). Washed the cells in PBS buffer, and then added DCFDA (10 mM) in serum-free medium at 37 °C for 30 min. The fluorescence of DCF in the ARPE-19 cells was detected with 525 nm as an emission wavelength and 485 nm as an excitation wavelength.

Malondialdehyde (MDA) was an end product of lipid peroxidation, which was detected to detect the levels of lipid peroxidation by a MDA assay kit (Sigma Chemical Co., USA). The results were shown to be nM/mg protein. WST-1 was used to detect the content of superoxide dismutase (SOD) in the lysis solution. The SOD WST-1 kit was purchased (Coherent, Santa Clara, CA, USA) and used based on the manufacturer’s instructions.

### Electron microscopy

Autophagosome in RPE, was characterized by transmission electron microscope FEI Tecnai G2 Spirit by a digital camera Morada. The cell was washed again and stained for 5 min in 2.5% aqueous uranyl acetate. The sample was dehydrated with graded alcohol and embedded in Epon resin. Images were acquired in the AC mode using a silicon tip with a typical resonance frequency of 300 kHz and a radius smaller than 10 nm. Autophagosome was identified under the microscope solely based on size and morphology.

### Treatment with inhibitors and western blot analysis

For the inhibition experiments, ARPE-19 cells were pre-incubated for 1 h with each inhibitor, such as lysosomal inhibitors NH4Cl, antioxidant N-acetyl cysteine (NAC) or HMGB1 inhibitor ethyl pyruvate (EP). Cells were washed in PBS and immediately lysed in RIPA buffer (Thermo, Carlsbad, CA, USA) supplemented with phosphatase/protease inhibitor cocktail (Thermo). The lysate was centrifuged at 15000 g for 20 min, and the supernatant was further used for analysis. Using bovine serum albumin as the standard to detect the protein concentration. The protein (40 μ g/well) was loaded and separated by SDS-PAGE and transferred to the nitrocellulose membrane. The membrane was coated with 5% non-fat skim milk in TBST (150 mM NaCl, 10 mM Tris-HCl [pH 8.0], 0.02% Tween20) for 1 h at 15 °C, and then probed overnight at 4 °C with primary antibodies. Primary antibodies used were LC3 (DAB; Sigma Chemical Co., USA), p62 (DAB; Sigma Chemical Co., USA) and β-actin (C DAB; Sigma Chemical Co., USA).

### Immunocytochemistry

Immunocytochemistry was also performed on ARPE-19 cells grown as monolayers on transwell plates, fixed in 4% paraformaldehyde. After extensive washing, cells were incubated either in rabbit polyclonal antibodies recognizing anti-HMGB1 (1:200; Invitrogen, Carlsbad, CA, USA) in blocking solution (0.5% Triton-X in tris buffered saline with 10% goat serum), and followed by Alexa Fluor 488 (1:500; Sigma Chemical Co., USA) as the secondary antibody. Staining was examined via fluorescence microscopy (Zeiss, USA) equipped with a digital camera.

### Statistical analysis

The data were presented as the mean ± SEM, and the data were compared using one-way analysis of variance (ANOVA). A *p*-value < 0.05 was considered statistically significant. All analyses were performed using a statistical software package (SPSS 15.0, Chicago, IL, USA).

## Results

### Intermittent high glucose induces significant oxidative stress and inhibits proliferative activity in ARPE-19 cells

**Fig. 1:**
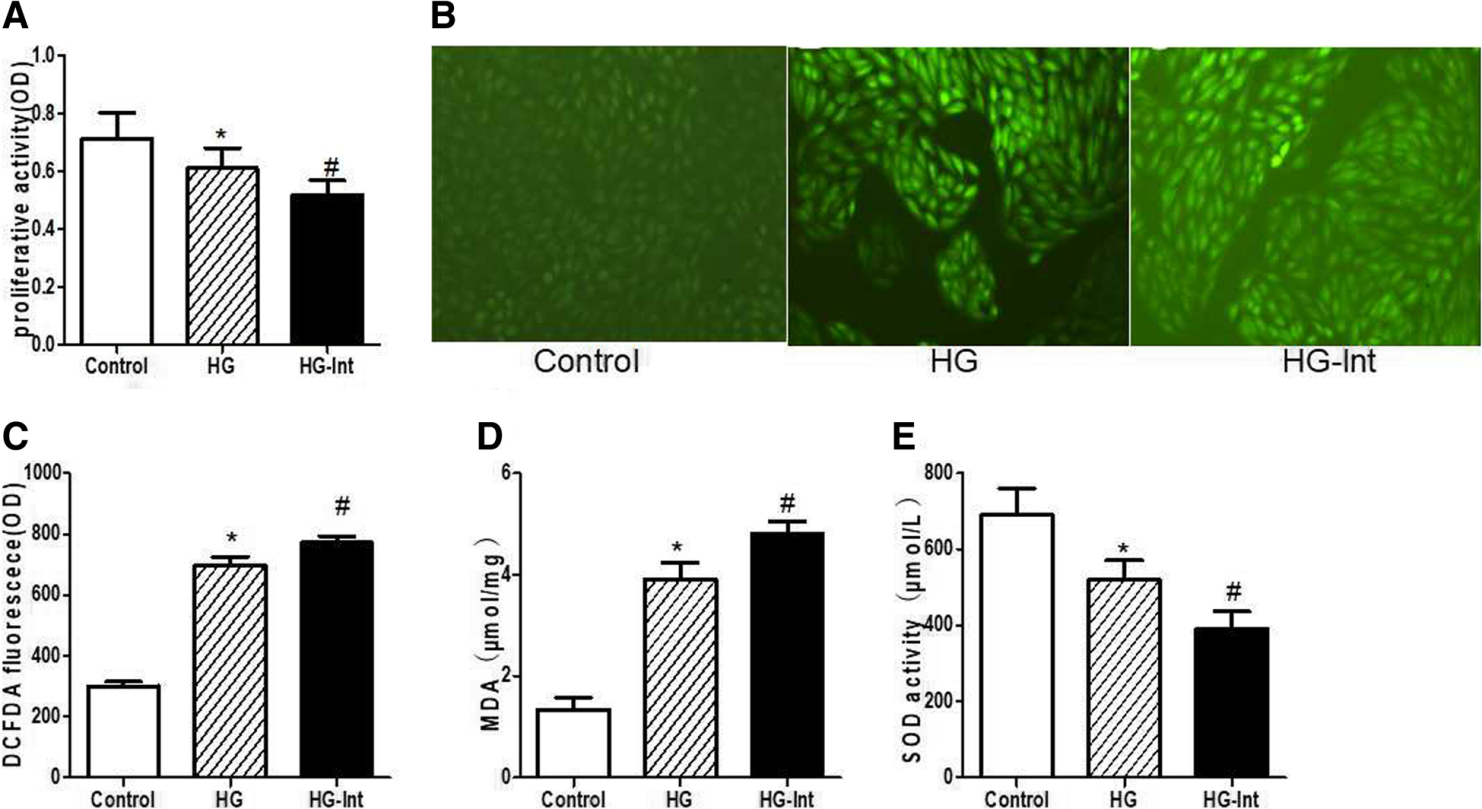
Intermittent high glucose induces significant oxidative stress and inhibits proliferative activity in ARPE-19 cells. **a**: Proliferative activity was decreased in both constant high glucose-treated cells and Intermittent glucose induced a more remarkable decrease in proliferative activity when compared to constant high glucose. **b**-**c**: Intermittent high glucose resulted in dramatic significantly higher fluorescence of cellular ROS marker DCFDA than constant high glucose and normal glucose. **d**: MDA concentrations of intermittent high glucose-treated cells was higher than that of constant high glucose and normal glucose. **e**: SOD concentrations in intermittent high glucose-treated cells was lower than that in constant high glucose and normal glucose. (*p < 0.05 vs. control, ^#^*p* < 0.05 vs. HG)

In this study, we exposed ARPE-19 cells to intermittent and constant high glucose to make an oxidative stress injury model. To prove that intermittent and constant high glucose could affect cell oxidative stress and survival, we first treated ARPE-19 cells with high glucose for 48 h and then detected cell proliferation by MTT assay. We found that proliferative activity was decreased in both constant high glucose-treated cells (0.613 ± 0.077 OD) and intermittent high glucose-treated cells (0.527 ± 0.045 OD) compared with cells exposed to normal glucose (0.714 ± 0.089 OD) (*p* < 0.05) (Fig. 1a). Intermittent glucose could greatly decrease the proliferative activity compared with constant high glucose (*p* < 0.05) (Fig. 1a), indicative of an inhibitory effect of proliferative activity on ARPE-19 cells. In following experiments using DCFDA, MDA and SOD assays, we found that intermittent high glucose resulted in dramatic significantly higher fluorescence of cellular ROS marker DCFDA (772.41 ± 20.352 OD) than constant high glucose (697.98 ± 27.798 OD) and normal glucose(300.04 ± 14.503 OD) (Fig. 1b, c). Similarly, MDA concentrations of intermittent high glucose-treated cells (4.818 ± 0.236 μmol/mg) was higher than that of constant high glucose (3.913 ± 0.317 μmol/mg) and normal glucose (1.338 ± 0.228 μmol/mg) (*p* < 0.05) (Fig. 1d). However, SOD concentrations in intermittent high glucose-treated cells (392.7 ± 47.5 μmol/L) was lower than that in constant high glucose (525.1 ± 51.2 μmol/L) and normal glucose (695.3 ± 73.4 μmol/L) (*p* < 0.05) (Fig. 1e), suggested that the oxidative stress increased in intermittent high glucose-treated cells. Taken together, these data suggested that intermittent high glucose could induce significant oxidative damage and inhibit proliferative activity than constant high glucose in ARPE-19 cells.

### Intermittent high glucose induces RPE autophagy and alters the expression pattern of autophagic markers

**Fig. 2:**
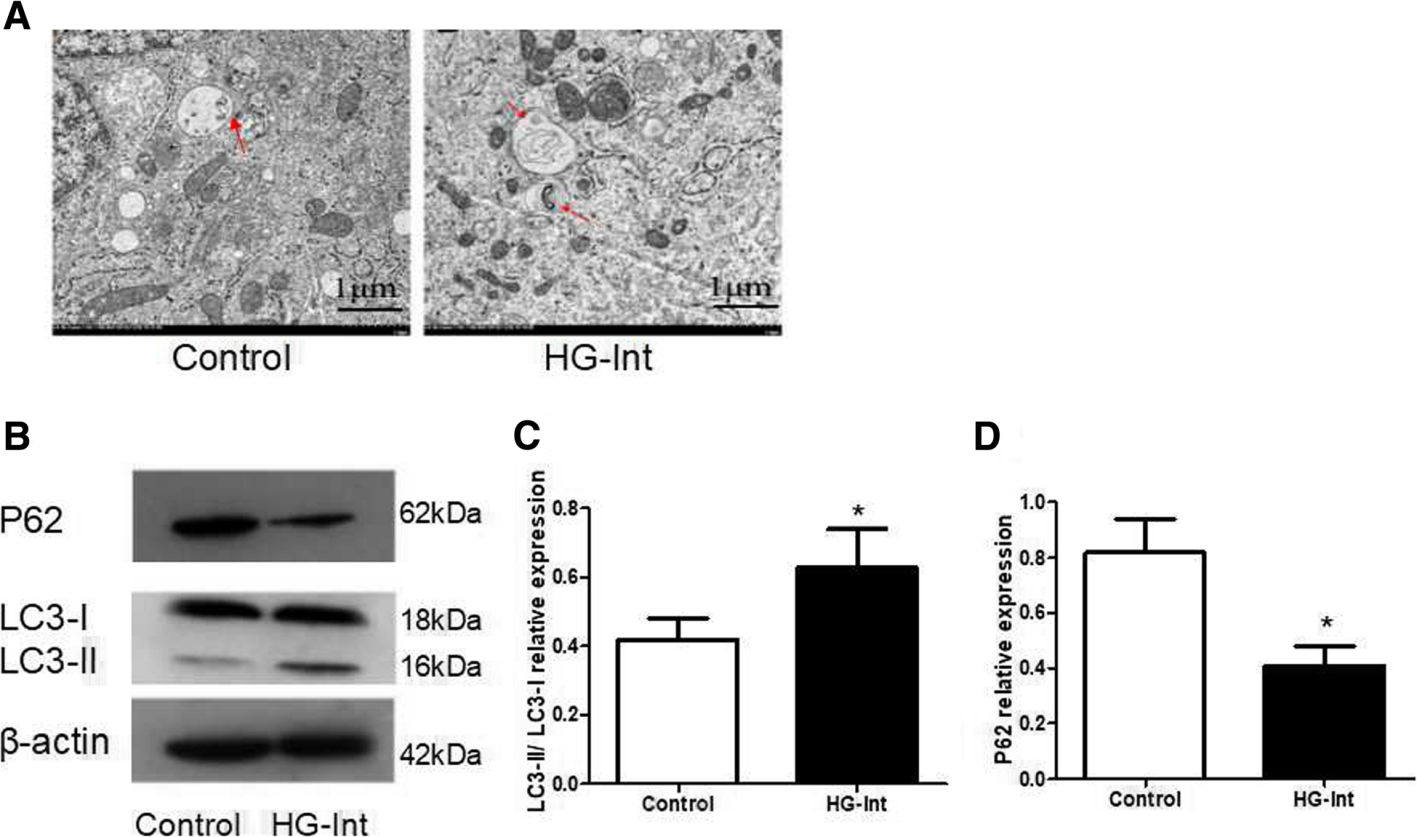
Intermittent high glucose induces RPE autophagy and alters the expression pattern of autophagic markers. **a**: Transmission electron microscopy examination revealed that intermittent high glucose lead to an increase in the number of double-membrane vacuoles, which was a typical of autophagosomes. **b**-**d**: Western blot results showed that intermittent high glucose lead to a significant increase in conservation of LC3-II. Intermittent high glucose resulted in a marked decrease in p62 protein expression. (*p < 0.05 vs. control, ^#^*p* < 0.05 vs. HG)

In order to determine the effect of intermittent high glucose on autophagy, ARPE-19 cells were seeded in the medium containing normal glucose (5 mM) or high glucose (25 mM) alternating every 3 h. Transmission electron microscopy examination revealed that intermittent high glucose lead to increasing of double-membrane vacuoles, which was a typical of autophagosomes (Fig. 2a). LC3 was processed from LC3-I to LC3-II, which could incorporate into vacuoles. In Fig. 2b, western blot results showed that intermittent high glucose lead to a significant increase in conservation of LC3-II (Fig. 2c), indicating that intermittent high glucose could induce RPE autophagy. P62 selectively incorporates into autophagosomes with the direct binding to LC3, which is ultimately degraded by autophagy [12]. Therefore, the amount of p62 was negatively correlated with autophagic activity. Our results showed that compared with LC3-II level, intermittent high glucose resulted in a marked decrease in p62 protein level (Fig. 2d). Collectively, the level of LC3-II/LC3-I and p62 suggests that intermittent high glucose induced autophagy in ARPE-19 cells.

### Effect of lysosomal inhibitors and antioxidant on intermittent high glucose-induced RPE autophagy and proliferative activity

**Fig. 3:**
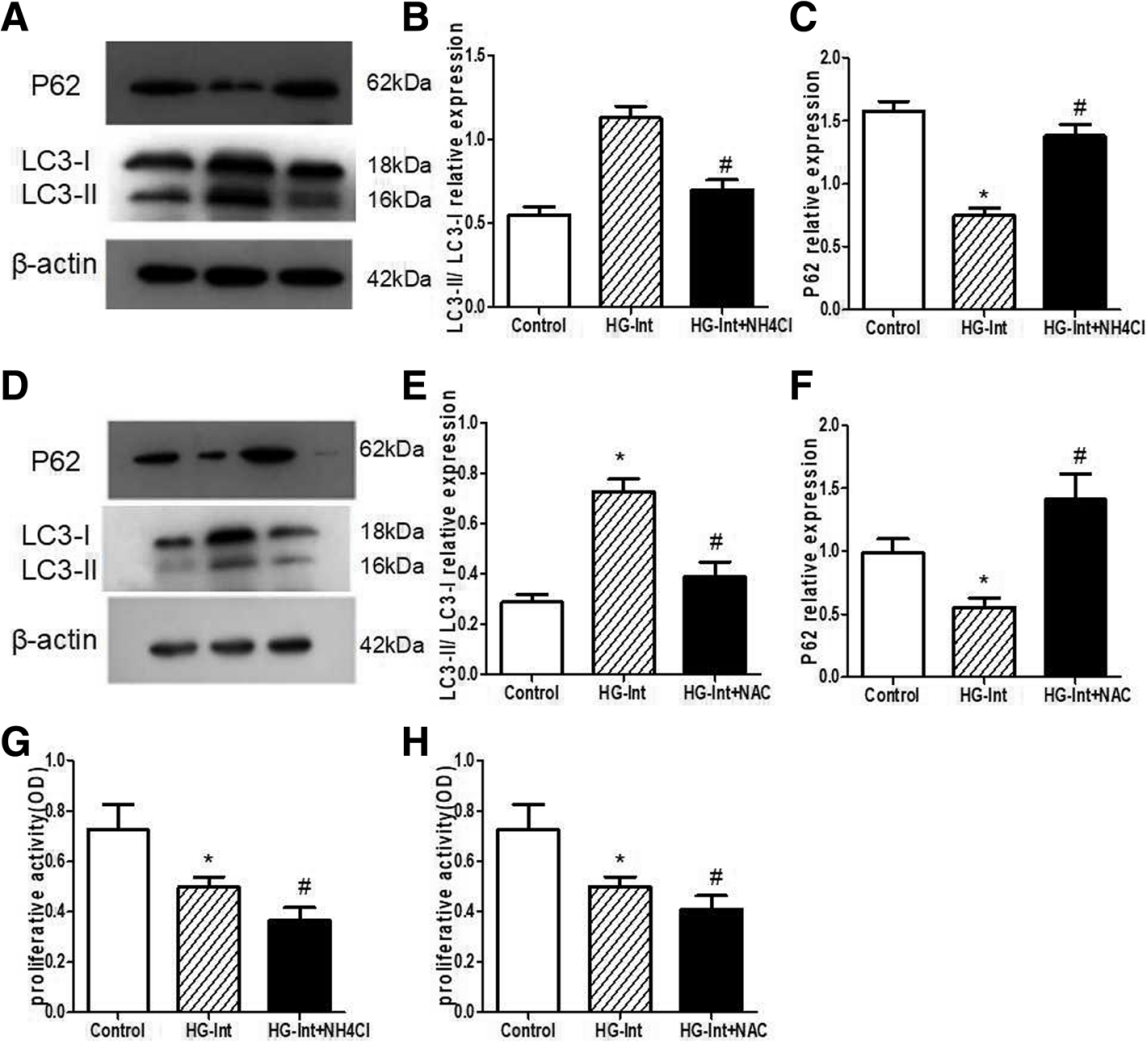
Effect of lysosomal inhibitors and antioxidant on intermittent high glucose-induced RPE autophagy and proliferative activity. **a**, **b**, **d**, **e**: Intermittent high glucose treatment in the presence of either NH4Cl or NAC decreased the level of LC3-II/LC3-I in ARPE-19 cells. **c**, **f**: Intermittent high glucose treatment in the presence of either NH4Cl or NAC resulted in a marked increase in p62 protein expression. **g**-**h**: Compared with the HG-Int group, intermittent high glucose treatment in the presence of either NH4Cl or NAC resulted in an obvious reduction in proliferative activity. (*p < 0.05 vs. control, ^#^*p* < 0.05 vs. HG)

NH4Cl as a lysosomal inhibitor can interrupt the lysosome-autophagosome fusion. N-acetylcysteine (NAC) was a molecule with antioxidant properties, which possessed a sulfhydryl group and acted as the source of cysteine to glutathione synthesis. To determine the effect of NH4Cl and NAC on intermittent high glucose-induced RPE autophagy, we measured the level of LC3-II/LC3-I in the presence of either NH4Cl or NAC. Intermittent high glucose treatment in the presence of either NH4Cl or NAC decreased the level of LC3-II/LC3-I in ARPE-19 cells (Fig. 3a, b, d, e), indicating that intermittent high glucose-induced increasing of LC3-II was blocked in the presence of either NH4Cl or NAC. Similaly, we found that compared with LC3-II/LC3-I level, p62 protein has an opposite expression trend. Intermittent high glucose treatment in the presence of either NH4Cl or NAC resulted in a marked increase in p62 protein level (Fig. 3c, f). Collectively, the level of LC3-II and p62 indicates that NH4Cl and NAC could inhibit intermittent high glucose induce autophagy in RPE.

We used MTT method to measure the RPE viability under intermittent high glucose stress. We showed that compared with the HG-Int group (0.498 ± 0.038 OD), intermittent high glucose treatment in the presence of either NH4Cl (0.365 ± 0.051 OD) or NAC (0.417 ± 0.053 OD) resulted in an obvious reduction in proliferative activity (*p* < 0.05) (Fig. 3g, h), suggesting that autophagy has a protective effect on RPE under intermittent high glucose stress.

### HMGB1 mediates intermittent high glucose-induced autophagy

**Fig. 4:**
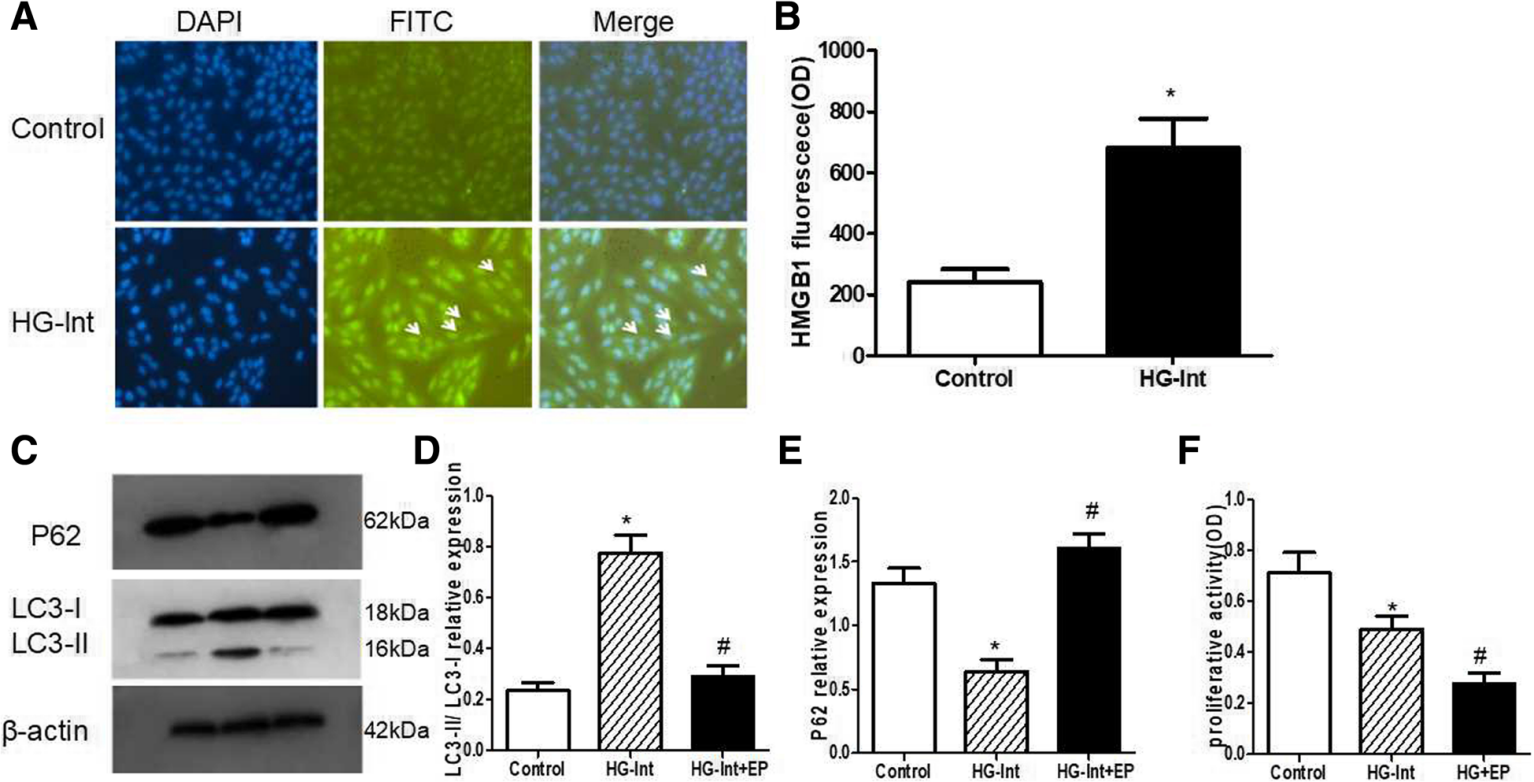
HMGB1 mediates intermittent high glucose-induced autophagy. DAPI was used to stain the nuclei. **a**-**b**: Immunohistochemistry showed that the normal glucose ARPE-19 cells were mainly expressed as nuclear localization of HMGB1. However, in ARPE-19 cells treated with intermittent high glucose, the proportion of HMGB1 in the cytoplasm was increased. **c**-**d**: EP could inhibite intermittent high glucose-induced LC3-II expression. **e**: EP could increase the expression of p62 protein under conditions of intermittent high glucose. **f**: Loss of HMGB1 under intermittent high glucose condition resulted in dramatic significantly reduction in proliferative activity than intermittent high glucose and normal glucose(*p < 0.05 vs. control, ^#^*p* < 0.05 vs. HG)

HMGB1 is a rich nuclear protein with pro-inflammatory activity dependent on its extra-nuclear function [13]. The distribution of HMGB1 was detected under intermittent high glucose condition, and ARPE-19 cells were stained with specific anti-HMGB1 antibody. ARPE-19 cells were mainly expressed as nuclear localization of HMGB1in the normal glucose condition. However, in ARPE-19 cells treated with intermittent high glucose, the proportion of HMGB1 in the cytoplasm was increased (Fig. 4a, b).

To detect if HMGB1 affects the expression of autophagy in response to intermittent high glucose condition, we examined the autophagic flux in the presence of HMGB1 inhibitor ethyl pyruvate (EP). Our results showed that EP could inhibite intermittent high glucose-induced LC3-II level (Fig. 4c, d), indicating an important role of HMGB1 in the regulation of intermittent high glucose-induced autophagy. In addition, EP could increase the level of p62 protein under conditions of intermittent high glucose (Fig. 4e), suggesting that the degradation is dependent upon HMGB1 mediated autophagy. Overall, our results suggest that HMGB1 is essential for autophagy induced by intermittent high glucose.

In following experiments using MTT assays, we found that loss of HMGB1 under intermittent high glucose condition resulted in dramatic significantly reduction in proliferative activity (0.277 ± 0.044 OD) than intermittent high glucose (0.492 ± 0.048 OD) and normal glucose (0.719 ± 0.078 OD) (*p* < 0.05) (Fig. 4f), suggesting that HMGB1 mediated autophagy has a protective effect on RPE under intermittent high glucose stress.

## Discussion

More evidences from cell culture and animal models show that oxidative stress is an important cause of RPE damage diabetic macular edema [14]. We found that intermittent high glucose induced autophagic flux in RPEs, which seems to have a protective effect on intermittent high glucose-induced RPE damage. It is possible that the repeated shift from normal glucose to high glucose during glucose fluctuation may lead to oxidative stress. Recent studies showed that autophagy plays a key regulator in the RPE and is a crucial role in protection against oxidative stress [15, 16]. In our study, we show that p62 is dramatically down-regulated under intermittent high glucose condition, which is related to enhanced autophagic flux in ARPE-19 cells and that lysosomal inhibitors NH4Cl or antioxidant NAC make ARPE-19 cells more susceptible to oxidative stress. Consistently, we demonstrated that NH4Cl or NAC decreases both p62 and autophagic flux. We also showed that autophagy may be required for preventing oxidative stress-induced injury in RPE under intermittent high glucose condition, while HMGB1 plays important roles in signaling for both autophagy and oxidative stress in ARPE-19 cells.

Autophagy is the conservative mechanism for the degeneration of cellular components in the cytoplasm [17]. It is shown that autophagy is a double-edged sword for cell physiology [18]. Autophagy acts as a cell survival mechanism under oxidative stress condition, and plays a key role in cell apoptosis [19]. p62/ Sequestosome1 (SQSTM1) is a multifunctional scaffold protein, which acts a key role in different cellular signaling pathways and plays an adaptive role in various cellular processes [20, 21]. It is shown that the inhibition of proteasoome caused elevated p62 level in RPE cells [16]. Our data indicates that intermittent high glucose induces expression of p62 in the ARPE-19 cells, which starts the autophagic pathway and considers as a protective response against high glucose induced oxidative stress. Previous researches have shown that high glucose promoted autophagy in endothelial cells by inhibiting PI3K/AKT signaling [7]. While in RPE cells, autophagy can prevent the oxidative damage caused by diabetes [22]. We have revealed that autophagy has a positive effect on RPEs under intermittent high glucose condition. In contrast, Yang F et al. showed an opposite phenomenon that inhibition of autophagy has a protective effect on high glucose-induced cardiac vascular endothelial cells injury [23]. These results emphasize the fact that autophagy may be either protective or harmful, which depends on cell types and cell environments. Therefore, the function of autophagy should be discussed separately under different pathological conditions [24].

HMGB1 protein is a chromatin-binding nuclear protein. It is a part of damage-related molecular patterns and has an important effect on oxidative stress response and cell death signals, involving autophagy and apoptosis [25]. Recent researches have showed that autophagy regulates the release of selective HMGB1 in endothelial cells that are destined to death [26, 27]. On the contrary, autophagy induced by exogenous HMGB1 could promote chemotherapeutic resistance in leukemia cells [28]. It is noteworthy that our experimental data show HMGB1 could be a regulator of high glucose-induced autophagy, the reasons as follow: 1) HMGB1 could regulate the turnover of LC3-II/ LC3-I. Under in which autophagy is increased, such as exposure to intermittent high glucose condition, HMGB1 regulated LC3-positive punctae is apparent. Correspondingly, without HMGB1 inhibited intermittent high glucose condition-induced LC3-positive punctae, suggesting HMGB1 regulated the LC3 reaction [29]. 2) HMGB1 can regulate the p62 autophagic degradation. The accumulation of p62 was found in the cells withoutHMGB1 deficient after oxidative stress, suggesting that the autophagic degradation or defects in autophagic degradation of p62 in the present of EP. It is important that continuous expression of p62 induced by autophagy defects is sufficient to alter NF-kB regulation, and then reduce the proliferation activity of ARPE-19 cells [30]. 3) Loss of HMGB1 in RPE cells promotes the production of ROS. The targeted inhibition of HMGB1 increased the production of ROS and reduced autophagy with oxidative stress, which indicates that the role of HMGB1 is the upstream of oxidative stress [31]. These results show a key signaling pathway that relates intermittent high glucose-induced RPE autophagy to dysregulation of HMGB1.

## Conclusions

Our data clearly indicate that activation of autophagy may be required for preventing oxidative stress-induced injury in RPE under intermittent high glucose condition by activation of HMGB1. Autophagy participates in oxidative stress induced by mitochondrial dysfunction. Foresti R et al. [32] found that RPEs in HG did not affect the activation of the Nrf2/heme axis but affected the oxidative and mitochondrial-dependent cellular functions. The inhibition of autophagy by blockage of HMGB1 suggests that HMGB1 may be suitable target for a protective therapy for DR. Understanding the role of intermittent high glucose condition-induced stimulation of autophagy in the RPE cells will provide new sight for the pathogenesis of DR.

## Abbreviations

DR: Diabetic retinopathy
HMGB1: High-mobility group box 1
MDA: Malonyldialdehyde
NAC: N-acetyl cysteine
ROS: Reactive oxygen species
RPE: Retinal pigmented epithelial
SOD: Superoxide dismutase
